# *NPHS2* Mutations: A Closer Look to Latin American Countries

**DOI:** 10.1155/2017/7518789

**Published:** 2017-07-12

**Authors:** Mara Sanches Guaragna, Anna Cristina G. B. Lutaif, Andréa T. Maciel-Guerra, Vera M. S. Belangero, Gil Guerra-Júnior, Maricilda P. De Mello

**Affiliations:** ^1^Center for Molecular Biology and Genetic Engineering (CBMEG), State University of Campinas (UNICAMP), Campinas, SP, Brazil; ^2^Integrated Center of Pediatric Nephrology (CIN), Department of Pediatrics, School of Medical Sciences (FCM), State University of Campinas (UNICAMP), Campinas, SP, Brazil; ^3^Department of Medical Genetics, School of Medical Sciences (FCM), Interdisciplinary Group for the Study of Sex Determination and Differentiation (GIEDDS), School of Medical Sciences (FCM), State University of Campinas (UNICAMP), Campinas, SP, Brazil; ^4^Interdisciplinary Group for the Study of Sex Determination and Differentiation (GIEDDS), Pediatrics Endocrinology, Department of Pediatrics, School of Medical Sciences (FCM), State University of Campinas, UNICAMP, Campinas, SP, Brazil

## Abstract

Nephrotic syndrome is one of the most common kidney pathologies in childhood, being characterized by proteinuria, edema, and hypoalbuminemia. In clinical practice, it is divided into two categories based on the response to steroid therapy: steroid-sensitive and steroid resistant. Inherited impairments of proteins located in the glomerular filtration barrier have been identified as important causes of nephrotic syndrome, with one of these being podocin, coded by* NPHS2* gene.* NPHS2* mutations are the most frequent genetic cause of steroid resistant nephrotic syndrome. The aim of this review is to update the list of* NPHS2* mutations reported between June 2013 and February 2017, with a closer look to mutations occurring in Latin American countries.

## 1. Introduction

In the high-throughput sequencing era, new candidate genes associated with monogenic and genetic heterogeneous diseases such as steroid resistant nephrotic syndrome (SRNS) are piling up [1–6]. However, mutations in the three main genes (*NPHS1*,* NPHS2*, and exons 8 and 9 of* WT1* gene) are still the most frequent molecular cause of SRNS in childhood and adolescence. More than 200* NPHS2* (OMIM ^*∗*^604766) gene mutations are registered in HGMD Professional 2017.1 (http://www.hgmd.cf.ac.uk) and 127 in the HGMD Public 2017.1 associated with familial and sporadic forms of SRNS.

SRNS is one of the most common kidney pathologies in childhood, being characterized by proteinuria, edema, and hypoalbuminemia. The most frequent renal histological feature associated with SRNS is focal segmental glomerulosclerosis (FSGS). Almost 40% of SRNS/FSGS children develop end-stage renal disease (ESRD) before adulthood and may receive a kidney transplant, with a 10 to 50% risk of recurrence of FSGS in the allograft kidney [7–9]. Although the pathogenesis of NS is not yet completely understood, much has been learnt about the glomerular filtration barrier (GFB) which is composed of three layers: the fenestrated capillary endothelial cells; the glomerular basement membrane (GBM); and the podocytes, specialized cells with interdigitating foot processes that are interconnected to form a slit diaphragm (SD) membrane, a multiprotein signaling complex that controls the ultrafiltration in this dynamic structure [10]. Nephrotic protein leakage may occur as a result from damage in one of these GFB components [11], although functional pathways specifically in the podocyte have revealed this cell as the key component of the pathogenesis of SRNS [5, 6, 12, 13].

More than 50 genes have been identified so far, associated with SRNS of congenital (0–3 months), infantile (4–12 months), childhood/adolescence (1–18 years), or adult onset [6]. In 2007, Hinkes et al. [14] screened the four genes* NPHS1* (OMIM ^*∗*^602716),* NPHS2* (OMIM ^*∗*^604766),* WT1* (OMIM ^*∗*^607102), and* LAMB2* (OMIM ^*∗*^150325) in a large European cohort of 89 children from 80 families with NS manifesting in the first year of life. They detected disease-causing mutations in one of the four genes in 66.3% of the families, 84.8% of congenital onset, and 44.1% of infantile onset. Seven years later, in 2014, Sadowski et al. [5] screened 27 genes associated with SRNS in 1783 families from an international cohort study (107 members from 30 countries). The eight larger contributing centers were Germany, Switzerland, Turkey, Egypt, Saudi Arabia, Los Angeles, Ann Arbor, and India but many other centers participated, including Argentina representing South America with 16 families. The main conclusion was that a monogenic cause was detected in 29.5% of the SRNS cases (0–25 years) in one of the 27 genes analyzed. After a detailed analysis of mutation distribution by gene and by age of onset,* NPHS2* mutations were the most frequent (5.7% to 12.7%) in patients with SRNS onset between 1 and 18 years old.

Mutations in* NPHS2* gene, located at 1q25-31, are the most common cause of SRNS in childhood and were first described by Boute et al. (2000) [15].* NPHS2* coding region encompasses 1,149 bp, has 8 exons, and encodes a 383-amino-acid protein with 42 kD, called podocin, which is expressed in fetal and mature kidney [15]. Podocin is predicted to have a hairpin-like structure, with both C- and N-terminal domains facing cytosol and one short transmembrane domain [16]. In addition to its role in anchoring nephrin and CD2AP (OMIM ^*∗*^604241) to the SD, podocin forms homoligomer complexes that bind with cholesterol in lipid rafts, where it may act as a scaffolding/targeting/signaling protein [17–19].* NPHS2* mutations initially found in autosomal-recessive inheritance familial cases [15] and further in sporadic SRNS cases [20] as well represent 40% and 6–17% of these cases, respectively [12, 21–23]. In 2013, Bouchireb et al. [24], in a detailed and complete review, presented a list of* NPHS2* mutations, polymorphisms, and variants of unknown significance published from October 1999 to September 2013. In that review they identified 25 novel pathogenic mutations in addition to the 101 already registered in the mutation database at that time. The mutations were distributed along the entire* NPHS2* gene, with no preferential hotspot.

The aim of this review is to update the list of* NPHS2* mutations reported in the last few years around the world, with a closer look to mutations occurring in Latin American countries.

## 2. *NPHS2* Mutations Overview

We searched for articles reporting* NPHS2* mutations associated with SRNS in childhood and adolescence that were published from June 2013 to February 2017. The key words* “NPHS2”*, “*NPHS2* mutations”, “podocin” and “steroid resistant nephrotic syndrome genetics” were used in PubMed databank. We further looked for variants/mutations that were not annotated in open-access databases such as public HGMD (http://www.hgmd.cf.ac.uk/ac/index.php) and GnomeAD Browser (http://gnomad.broadinstitute.org) or in Leiden Open Variation Database (https://www.lovd.nl/NPHS2). For nonsynonymous variants, we classified them as deleterious or benign according to in silico prediction tools (PolyPhen-2 and SIFT) [25, 26]. For splicing variants, we performed splice-site prediction by BDGP neural network [27].

Thirty-nine variants, among them 25 missenses, four nonsenses, three splice-sites, four frameshifts, and three in the promoter region were published from June 2013 to February 2017 in a total of 109 out of 829 SRNS patients in many countries: China (Wang et al., 2017) [28]; India (Jaffer et al., 2014; Dhandapani et al. 2017; Ramanathan et al. 2017) [29–31]; Italy (Benetti et al., 2013) [32]; Iran (Basiratnia et al., 2013) [33]; United Kingdom (Jain et al., 2014) [34]; United States of America (Laurin et al., 2014; Phelan et al., 2015) [35, 36]; Poland (Kuleta et al., 2014) [37]; Finland (Suvanto et al., 2016) [38]; Saudi Arabi (Kari et al., 2013) [39]; Japan (Ogino et al., 2015) [40]; Mexico (Carrasco-Miranda et al., 2013) [41]; Chile (Azocar et al., 2016) [42]; and Brazil (Guaragna et al., 2015) [43]. Ten out of those 39 mutations were unique and had not been annotated in public HGMD (http://www.hgmd.cf.ac.uk/ac/index.php) or in GnomeAD Browser (http://gnomad.broadinstitute.org) or in the Leiden Open Variation Database (https://www.lovd.nl/NPHS2): six were missenses, three were located in splice-site regions, and two were frameshifts (Table 1). We searched GnomaAD Browser for all the variants compiled in Table 1 as well as for other variants such those reported in Mexico (p.Leu142Pro), Chile and Brazil (p.Ala284Val), and Brazil (p.Val260Glu), observing frequency, racial ethnicity, and geographical provenience. Only p.Ala284Val and p.Val260Glu were registered at GnomaAD Browser, but no allele counted was from Latin America population for both of them.

## 3. Missense Mutations

Four out of the five missenses were described in homozygosity in South Indian SRNS patients (p.Ser46Pro, p.Leu167Pro, p.Pro175Ser, and p.Pro316Ser) [29, 30]. The fifth missense, p.Leu139Arg, was identified in two Mexican children with NS, one SRNS, and one SSNS [41]. As in silico predictions were not performed for those variants in their original publications, we investigated their pathogenicity by predictive tools available, such as SIFT and PolyPhen-2. Both p.Leu139Arg and p.Pro316Ser variants were predicted as damaging by SIFT and PolyPhen-2 (Table 1). At the moment, these five missense variants should be considered as variants of unknown significance and only after proper functional studies they can be associated with SRNS.

## 4. Splice-Site Mutations

Three splice-site mutations that were not registered in any of the three searched databases have been identified (Table 1). The homozygous splice-site mutation c.451+3A>T whose effect on podocin protein was evaluated by renal mRNA analysis demonstrated exon 3 skipping that led to a premature termination codon (p.Val128Phefs^*∗*^28). This mutation was originally identified by Benetti et al. [32] in an Italian girl with SRNS. Either c.535-1G>A or c.738+2T>C were described by Wang et al. [28] in compound heterozygosis with another known* NPHS2* mutation in two nonrelated SRNS Chinese children. They evaluated the conservation of variant sites using PhyloP Primates tool that resulted in scores of 4.481 and 3.839 for c.535-1G>A and c.738+2T>C, respectively, indicating high degree of conservation at these sites. They also evaluated those variants using Mutation Taster that classified them as damaging. Nevertheless, they did not use the BDGP Splice Site Prediction by Neural Network in silico tool to predict the splicing recognition sites; therefore, we performed this analysis and the resulting prediction is shown in Table 1.

## 5. Frameshift Mutations

Two small deletions were described: one (p.Ser329 = fs^*∗*^14) was found in heterozygosis in five SRNS individuals from the same Finnish family, with early-onset, slow progression, and dominant inheritance pattern [38]; the other (p.Lis239Argfs^*∗*^13) was identified by our group in two Brazilian sisters with early-onset SRNS in association with the p.Val260Glu missense [43].

## 6. *NPHS2* Mutations in the World with a Closer Look to Latin America

Population studies from different countries, mainly from Europe, South Asia, and North America, revealed that the prevalence of* NPHS2* mutations in children with SRNS may vary according to ethnicity. It appears to be frequent among Americans and Turkish [21, 44] (26% and 24.7%, resp.) but not as frequent among Greek [45], Chinese [28, 46], Indian [47], Japanese [40, 48], Pakistani [49], and Korean [50] patients (9%, 4.3%, 4%, 4%, 3.4%, and 0%, resp.). Recently a large multicentric study was performed with samples from 1783 SRNS families from eight contributing centers in which twenty-seven SRNS associated genes were sequenced. Disease-causing mutations were identified in different genes; however, mutations in* NPHS2 *were more frequent [5, 13]. Some of them, with a high frequency in particular geographical regions, are considered as founder alleles for* NPHS2*: p.Arg138Gln and p.Gly140Aspfs^*∗*^41 are predominant in Europe; p.Pro118Leu in Turkey; p.Val180Met in North Africa; p.Arg138^*∗*^ in Israel and Arabian countries; p.Val260Glu in Oman, Arabia; and p.Met1? and Asn199Lysfs^*∗*^14 in Egypt [5, 15].

**Figure 1:**
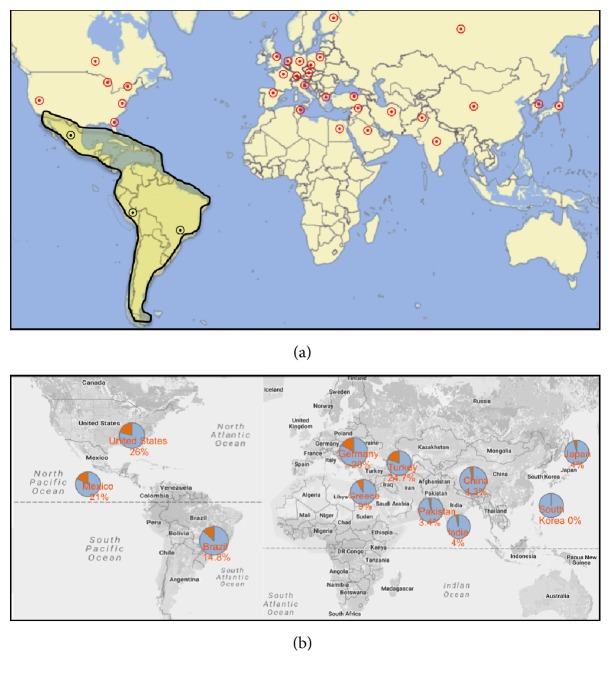
(a) Map of the main countries where* NPHS2 *mutations have been studied so far in SRNS cohorts around the world (red circle marks). Latin America is highlighted in darker yellow, with the three main centers (Mexico, Chile, and Brazil), where* NPHS2* mutations had been published represented by black circle marks. (b) Pye charts representing the percentage of* NPHS2 *mutations (in orange) found in some countries from South Asia, East Asia, Europe, North America, and South America.

The contribution of Latin American countries to genetic studies in SRNS is scarce. Searches for* NPHS2* mutations had been performed mainly in three countries: Mexico, Chile, and Brazil (Figure 1). In Mexico, only the 3rd exon of* NPHS2* was sequenced in eight SRNS and five SSNS children [41]. The heterozygous p.Leu139Arg variant was identified in two patients, one SRNS and one SSNS; therefore, it was considered as a variant of unknown significance. In Chile, Azocar et al. [42] performed a molecular study in SRNS children and found* NPHS2* mutations in 21%. The mutations identified were homozygosis for p.Pro341Ser in one patient and compound heterozygosis for p.Arg229Gln and p.Ala284Val in six patients [42, 51]. In Brazil, our group performed the molecular analysis of* NPHS2* in 27 SRNS children and identified disease-causing mutations in 14.8%. We identified the following associations: the [p.Ala284Val];[p.Arg229Gln] and [p.Ala284Val(;)p.Arg229Gln] in two sporadic unrelated patients with late-onset SRNS; the [p.Glu310Lys];[p.Arg229Gln] association in one sporadic patient with early-onset SRNS, and the [p.Lis239Argfs^*∗*^13(;)p.Val260Glu] in a familial case also with early-onset SRNS [43]. Although performed in small samples, those studies suggest that the [p.Ala284Val];[p.Arg229Gln] association is frequent in South American countries. Actually, 13 out of 14 South American families evaluated by Machuca et al. [52] also carried the [p.Ala284Val];[p.Arg229Gln] association, with one-half presenting the adult onset form of the disease. The p.Val260Glu variant is worth mentioning in this group, which is already considered as a founder allele in Oman, Arabia [5], and also identified in one of our familial cases. We are not aware of an Arabian ancestrality of this family, but given the highly miscegenated nature of the Brazilian population, we cannot exclude this possibility.

This review aimed to give a new perspective to Nephrotic Syndrome in Latin American countries, emphasizing the importance of implementing the molecular evaluation of NS, especially investigating mutations on those genes more frequently associated with SRNS in this region. The molecular characterization of SRNS in childhood and adolescence is relevant to guide further treatment, since patients bearing* NPHS2 *mutations may be spared of the undesired side effects of corticosteroids. Additionally, living donor transplantation might be considered since SRNS patients with homozygous or compound heterozygous mutations in* NPHS2 *have reduced risks for recurrence of FSGS after renal transplant compared with children without mutations.

## Figures and Tables

**Table 1:** *NPHS2* variants described from June 2013 to February 2017^*∗*^.

Exon/intron	Nucleotide change	Amino acid change	In silico prediction consequence		Ref.	Country
			PolyPhen/score^a^	SIFT/score^b^		
Missense						
1	c.133T>C	p.Ser46Pro	B/0.0	T/0.28	[29]	India
3	c.415T>A	p.Leu139Arg	P/0.967	D/0.0	[41]	Mexico
4	c.500T>C	p.Leu167Pro	P/1.0	D/0.0	[29]	India
4	c.523C>T	p.Pro175Ser	B/0.0	D/0.0	[29]	India
8	c.946C>T	p.Pro316Ser	P/0.976	D/0.02	[30]	India

Exon/intron	Nucleotide change	Amino acid change	Splice-site prediction by neural network		Ref.	Country
			Score normal sequence	Score mutant sequence		

Splice-site						
Intron 3	c.451+3A>T	—	0.54	0	[32]	Italy
Intron 4	c.[53]5-1G>A	—	0.99	0.54	[28]	China
Intron 5	c.738+2T>C	—	0.90	0	[28]	China

Exon/intron	Nucleotide change	Amino acid change	Consequence		Ref.	Country

Frameshift						
5	c.714delG	p.Lis239Argfs^*∗*^13	D		[43]	Brazil
8	c.988_989delCT	p.Ser329 = fs^*∗*^14	D		[38]	Finland

^*∗*^The variants presented in this table were not annotated in the public HGMD (http://www.hgmd.cf.ac.uk/ac/index.php) (access date May 31) or in the GnomeAD Browser (http://gnomad.broadinstitute.org) (access date May 31) or in the Leiden Open Variation Database (https://www.lovd.nl/NPHS2) (access date May 31); ^a^PolyPhen: 1.000 = probably damaging (P); 0.5000 = possibly damaging (PO); 0.000 = benign (B). ^b^SIFT: ≤0.05 = damaging (D); >0.05 = tolerated (T).
